# Integrative Network Pharmacology, Molecular Docking, and In Vitro Insights Into the Mechanism of Finger Citron for Non‐Alcoholic Fatty Liver Disease

**DOI:** 10.1002/fsn3.71695

**Published:** 2026-04-03

**Authors:** Qingpeng Li, Huilong Sun, Xiaoling Chen, Chunyang Chen, Yongsheng Chen, Hui He, Fan Yang, Dan Wang, Lin Zhou

**Affiliations:** ^1^ School of Life Sciences and Biopharmaceutics Guangdong Pharmaceutical University Guangzhou China; ^2^ School of Pharmacy Guangdong Pharmaceutical University Guangzhou China; ^3^ Guangdong Provincial Key Laboratory for Research and Evaluation of Pharmaceutical Preparations Guangdong Pharmaceutical University Guangzhou China; ^4^ The First Affiliated Hospital/The First Clinical Medicine School of Guangdong Pharmaceutical University Guangdong Pharmaceutical University Guangzhou China; ^5^ Department of Food Science and Engineering Jinan University Guangzhou China

**Keywords:** finger citron, HepG2 cells, molecular docking, network pharmacology, nonalcoholic fatty liver disease

## Abstract

Finger citron (FC), a traditional plant with both pharmacological activity and food‐grade safety, is considered to possess potential for the improvement of non‐alcoholic fatty liver disease (NAFLD). However, the bioactive constituents responsible for this effect and their underlying mechanisms remain incompletely understood. In this study, an integrative approach combining network pharmacology, molecular docking, and in vitro validation was employed to elucidate the mechanisms by which FC exerts anti‐NAFLD effects. Network pharmacology analysis identified 62 bioactive compounds targeting 306 liver injury‐related genes, among these, 3,4,7‐trimethoxycoumarin (TMC) was selected for further investigation. Gene Ontology (GO) enrichment revealed that FC‐mediated effects were significantly associated with protein phosphorylation, inflammatory responses, and protein kinase activity, whereas Kyoto Encyclopedia of Genes and Genomes (KEGG) pathway enrichment highlighted key involvement of the PI3K‐Akt and MAPK signaling pathways. Molecular docking demonstrated high binding affinities between TMC and key target proteins including IL‐6, TNF‐α, albumin (ALB), AKT1, and STAT3. The successful synthesis of TMC was confirmed via ^1^H‐NMR and MS analyses. In vitro studies revealed that TMC effectively reduced lipid accumulation and oxidative stress induced by free fatty acids in HepG2 cells. Furthermore, RT‐qPCR analysis showed that TMC modulated the mRNA expression of key target genes and lipid metabolism‐associated genes, including CPT2, and APOC2, thereby substantiating the mechanistic basis of its therapeutic action. Collectively, these findings highlight the therapeutic potential of FC against NAFLD and provide a scientific basis for its further development as a promising NAFLD intervention.

## Graphical abstract

An integrative strategy combining network pharmacology, molecular docking, and in vitro cellular experiments was employed to elucidate the anti‐NAFLD mechanism of FC. A total of 62 bioactive compounds and 306 common target genes were identified, followed by PPI, GO, and KEGG analyses to screen key targets and pathways. Among the candidate compounds, TMC was selected for validation. Molecular docking demonstrated strong interactions between TMC and major target proteins. In HepG2 cells, TMC effectively inhibited free fatty acid‐induced lipid accumulation and oxidative stress, whereas regulating the expression of core target genes, thereby supporting the therapeutic potential of FC against NAFLD.

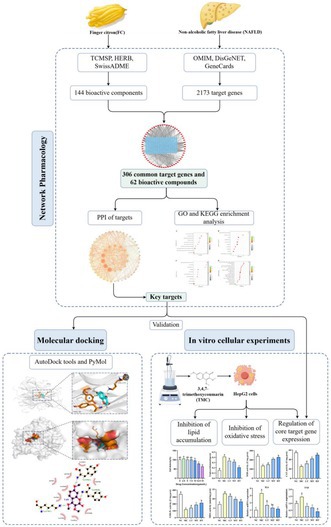

**Abbreviations**
CAT: catalase
CCK‐8: cell counting kit‐8
FC: finger citron
FFA: free fatty acids
GO: Gene Ontology
GSH‐PX: glutathione peroxidase
KEGG: Kyoto Encyclopedia of Genes and Genomes
MDA: malondialdehyde
NAFLD: non‐alcoholic fatty liver disease
PPI: protein–protein interactions
SOD: superoxide dismutase
TC: total cholesterol
TG: triglyceride
TMC: 3,4,7‐trimethoxycoumarin

## 1 Introduction

Chronic liver disease involves progressive hepatocellular injury, which promotes a pro‐inflammatory microenvironment in various hepatic cell populations. This process can ultimately lead to fibrosis, cirrhosis, and hepatic failure (Taru et al. 2024). Among these conditions, non‐alcoholic fatty liver disease (NAFLD) has become the most prevalent form of chronic liver disease, representing a substantial global health burden. Current epidemiological estimates indicate that NAFLD affects roughly 32% of adults worldwide, with its prevalence expected to rise sharply by 2030 (Du et al. 2019; Teng et al. 2023). Current therapeutic options are largely limited to lifestyle interventions, pharmacological treatments, and metabolic surgery. However, lifestyle changes demand sustained behavioral compliance, which is often difficult to maintain over the long term. Pharmacologic options remain limited, with no approved agents specifically targeting NAFLD, and existing drugs frequently exhibit unsatisfactory clinical efficacy or adverse side effects. Although bariatric surgery has demonstrated clinical benefit in selected patients, its application is constrained by surgical risks and potential complications. Therefore, NAFLD has emerged as a major liver disorder of global concern (Rong et al. 2022; Wei et al. 2024; Guo et al. 2022).

Natural medicines have garnered growing interest as potential therapeutics for NAFLD due to their complex composition, synergistic multi‐target activities, and favorable safety profiles (Song et al. 2025). Finger citron (FC, *Citrus medica* L. var. *sarcodactylis* Swingle), a traditional food‐medicine homologous plant, combines food‐grade safety with medicinal potential and has attracted attention for its development as a functional food and natural therapeutic agent (Ma, Tang, et al. 2021). Phytochemical analyses have shown that FC extracts are abundant in bioactive constituents—including neolignans, flavonoids, and coumarins—that display a broad spectrum of biological activities such as lipid‐lowering, antioxidant, anti‐aging, and anti‐tumor effects (Ma, Tang, et al. 2021; Wang et al. 2023; Luo et al. 2020; Ma, Wei, et al. 2021). Among these constituents, coumarins have attracted particular interest due to their unique benzopyrone core structure, low molecular weight, ease of structural modification, favorable pharmacokinetic properties, and wide distribution in plants (Annunziata et al. 2020). Increasing evidence suggests that coumarins exert significant lipid‐regulating, anti‐inflammatory, anti‐diabetic, and hepatoprotective effects by mitigating oxidative stress, enhancing insulin sensitivity, and promoting lipid turnover, thereby contributing to lipid metabolic homeostasis (Al Mouslem et al. 2022; Wang et al. 2025). Their pleiotropic actions align with the holistic pharmacological profile of FC, indicating that coumarins may represent key bioactive compounds mediating its therapeutic potential against NAFLD. Nevertheless, despite growing interest, the specific bioactive compound(s) responsible for the potential anti‐NAFLD effects of FC and their regulatory pathways remain to be identified. Therefore, further investigation is warranted to elucidate the specific bioactive compounds and underlying pathways involved in its hepatoprotective activity.

The integrated application of systems biology approaches—including network pharmacology and molecular docking simulations—offers a comprehensive and multidimensional strategy for elucidating the molecular mechanisms of traditional Chinese medicine. Network pharmacology enables the systematic exploration of interactions between bioactive compounds and disease‐associated targets by integrating Gene Ontology (GO) functional annotation with Kyoto Encyclopedia of Genes and Genomes (KEGG) pathway enrichment analyses (Li et al. 2023). Molecular docking utilizes flexible or semi‐flexible algorithms to simulate the optimal binding conformations between bioactive compounds and target proteins, thereby providing quantitative assessments of binding affinities (Ye, Li, and Hu 2021; Sun et al. 2025). In this study, we adopted an integrated strategy incorporating network pharmacology, molecular docking, and in vitro cellular experiments to elucidate the potential mechanisms underlying the therapeutic effects of FC against NAFLD. This comprehensive strategy aims to establish both theoretical and experimental foundations for the development of innovative natural product‐derived therapeutics.

## 2 Materials and Methods

### 2.1 Materials

HepG2 cells were obtained from the American Type Culture Collection (ATCC) strain preservation center. Culture media components, including DMEM high‐glucose medium, fetal bovine serum (FBS), and 1% penicillin–streptomycin (P/S), were sourced from Gibco (Grand Island, NY, USA). Oleic acid and palmitic acid were purchased from MedChemExpress LLC (NJ, USA). Cell counting kit‐8 (CCK‐8) was purchased from BeyoClick (Shanghai, China). Total cholesterol (TC) assay kit, triglyceride (TG) assay kit, catalase (CAT) assay kit, superoxide dismutase (SOD) assay kit, glutathione peroxidase (GSH‐Px) assay kit, and malondialdehyde (MDA) assay kit were purchased from Nanjing Jiancheng Bioengineering Institute (Nanjing, China). TRIzol reagent, Triton X‐100 lysis buffer, and the Bicinchoninic Acid Protein Assay kit were purchased from Shanghai Beyotime Biotechnology Co. Ltd. (Shanghai, China). HyperScript RT SuperMix for qPCR kit and HotStart 2X Green qPCR Master Mix kit were purchased from APExBIO Technology LLC (TX, USA). All other chemicals and reagents were of analytical grade and purchased from local suppliers.

### 2.2 Identification of Bioactive Components

Potential bioactive compounds in FC were retrieved from the TCMSP database (Ru et al. 2014) (https://tcmsp‐e.com/load_intro.php) and the HERB database (Fang et al. 2021) (http://herb.ac.cn/). Subsequently, key ADME parameters of the compounds, including oral absorption, lipophilicity, water solubility, and CYP450 enzyme inhibition, were analyzed using the SwissADME platform (Daina et al. 2017) (www.swissadme.ch). The target genes of the selected compounds were predicted through the Swiss Target Prediction platform (Daina et al. 2019) (www.swisstargetprediction.ch), with a screening criterion of probability > 0. We employed “liver injury” as the disease keyword in order to encompass a broad spectrum of liver damage mechanisms and maximize target coverage given the complexity of NAFLD. The targets closely related to liver injury were obtained from the OMIM database (Amberger et al. 2015) (https://www.omim.org), DisGeNET database (Piñero et al. 2020) (https://disgenet.com), and GeneCards database (Stelzer et al. 2016) (https://www.genecards.org). Finally, the intersecting targets of FC and liver injury were analyzed using Venny 2.1.0 (Bardou et al. 2014) (https://bioinfogp.cnb.csic.es/tools/venny/). Network topology analysis was performed using Cytoscape 3.10.1 in combination with the CentiScaPe 2.2 plugin to identify key targets.

### 2.3 Analysis of PPI Network

The protein–protein interactions (PPI) network of FC's predicted targets for liver injury was constructed using the STRING database (Szklarczyk et al. 2021) (https://string‐db.org/), with species restricted to “ *Homo sapiens* ”.

### 2.4 GO and KEGG Pathway Enrichment Analyses

GO and KEGG pathway enrichment analyses were performed using the DAVID database (Sherman et al. 2022) (https://davidbioinformatics.nih.gov/), focused on “ *Homo sapiens* ” gene annotations. Statistical significance was defined as *p* ≤ 0.05 with a false discovery rate (FDR) threshold of ≤ 0.05.

### 2.5 Molecular Docking

Key target proteins with highest degree values in the PPI network were selected as receptors, and the key bioactive component was designated as the ligand for molecular docking analyses. Prior to docking, both ligands and proteins underwent standardized preparation. Hydrogen atoms were added to the ligand structures, followed by energy minimization using the General Amber Force Field (GAFF) in Avogadro 1.2.0, applying 500 steps of the steepest descent algorithm to eliminate unfavorable steric conformations. Protein crystal structures were selected based on resolution, completeness, and biological relevance. Crystallographic water molecules were removed, and all hydrogen atoms were added before docking analysis (Mandal et al. 2009). The crystal structures of the key target proteins were obtained from the Uniprot database (UniProt Consortium 2025) (https://www.uniprot.org) and the Protein Data Bank (PDB) database (Berman et al. 2000) (http://www.rcsb.org). Docking grids and simulations were performed using AutoDock Tools 1.5.7 software (Morris et al. 2009). Blind docking, refined docking, and re‐docking to native ligand pose were employed for docking tests and validation. The grid center for the docking search was established based on literature references (Hetényi and van der Spoel 2011). The docking parameters were set as follows: the number of genetic algorithm (GA) runs was set to 100, with a maximum of 4,000,000 evaluations and 40,000 generations, whereas all other GA parameters were kept constant. The binding poses with the lowest binding energy obtained from blind docking were chosen for targeted docking to confirm the results. Visualization of docking results was performed using PyMol 1.5 software (Ye, Luo, et al. 2021).

### 2.6 Synthesis of 3,4,7‐Trimethoxycoumarin

**FIGURE 1:**
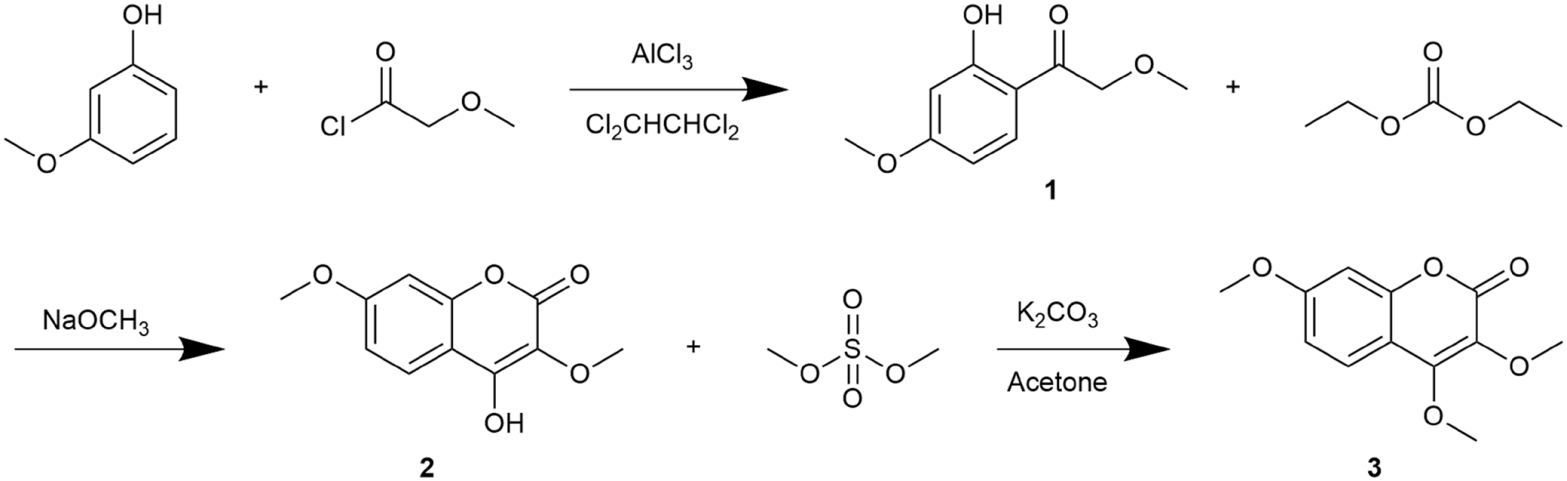
Synthesis process of TMC (compound **3**).

3,4,7‐trimethoxycoumarin (TMC) was synthesized according to the previous reports (Lee and Park 2012; Ahluwia and Prakash 1975) with some revision (Figure 1). First, aluminum chloride (10 mM) was added into 40 mL 1,1,2,2‐tetrachlorothane. Then, 3‐methoxyphenol (10 mM) and methoxyacetyl chloride (10 mM) were added into the mixture, respectively while stirring. After that, the mixture was stirred under room temperature overnight. After reaction, 30 mL of 1 M HCl was added into the mixture, followed by extracting with dichloromethane. The extracted solution was washed with sodium hydrogen carbonate. Then, the solvent was removed, and crystal (compound **1**) was collected for the following reaction. One gram crystal (compound **1**) was then dissolved with 20 mL of diethyl carbonate and 0.4 g of sodium methoxide was added into it. The solution was maintained at 60°C with continuous stirring for 4 h, after which it was cooled to room temperature and diluted with water. The resulting solid (compound **2**) was collected for subsequent reactions. One gram of compound **2** was dissolved in 50 mL of acetone, followed by the addition of 2 g of anhydrous potassium carbonate. Subsequently, 0.5 mL of dimethyl sulfate was introduced into the mixture, which was then maintained at 50°C for 4 h. Upon completion, potassium carbonate and the solvent were removed, and the product was subjected to recrystallization and silica gel column purification to yield compound **3**. ^1^H NMR (400 MHz, DMSO‐d6) δ 3.77 (s, 3H, OCH3 at C‐7), 3.82 (s, 3H, OCH3 at C‐4), 4.25 (s, 3H, OCH3 at C‐3), two doublets at 6.94, 6.96 (corresponding to C‐6 and C‐8 protons) and 7.63 (d, 1H at C‐5) ppm. Ms. *m*/*z* (%) 259 (100).

### 2.7 Cell Culture and Stimulation

HepG2 cells were maintained in a humidified incubator at 37°C with 5% CO_2_, using DMEM high‐glucose medium supplemented with 10% FBS and 1% P/S. Cells in the logarithmic growth phase were used for experiments when the confluence reached approximately 90%.

Building upon screening results and the protocol described by Zhou et al. (2018), HepG2 cells were incubated with 0.5 mM free fatty acids (FFA) at a 2:1 M ratio of oleic acid to palmitic acid, for 24 h under standard culture conditions (37°C, 5% CO_2_). This treatment established an in vitro NAFLD model for investigating the potential mechanisms of TMC intervention. For the stimulation experiments, cells were treated for 24 h with either 0.1% DMSO (negative control, NC), 0.5 mM FFA alone (model control, MC), or 0.5 mM FFA in combination with varying concentrations of TMC.

### 2.8 CCK‐8 Assay

HepG2 cells were seeded in 96‐well plates at a density of 1 × 10^4^ cells per well. Following 24 h of incubation, the cells were exposed to different concentrations of TMC (0–15 μM) for an additional 24 h. Subsequently, a 10% CCK‐8 solution, prepared in serum‐free medium, was added to each well, and the plates were incubated at 37°C for 1 h. Absorbance was measured at 450 nm using an ELX800 microplate reader (BioTek, AZ, USA), and cell viability was calculated from the absorbance values.

### 2.9 Determination of TC and TG Levels

Treatments were administered as specified in the figure legend. TC and TG levels were measured according to the protocol of the kits. Additionally, the protein concentrations were measured, and biochemical results were normalized to protein content.

### 2.10 Assessment of Oxidative Stress Levels

SOD, CAT, GSH‐Px, and MDA levels were assessed following the manufacturer's protocol.

### 2.11 RT‐qPCR Analysis

Total RNA was extracted from the HepG2 cells using the TRIzol reagent. The cDNA was synthesized using HyperScript RT SuperMix for qPCR kit. The relative mRNA expressions of IL6, TNF‐α, albumin (ALB), AKT1, STAT3, CPT2, and APOC2 were quantified using Reverse Transcription quantitative PCR (RT‐qPCR) on an ABI QS1 Plus system (Applied Biosystems, CA, USA). GAPDH served as the reference gene, and the relative mRNA expression levels were calculated using the $2^{-∆∆C_{T}}$ method. The primer sequences are listed in Table S1.

### 2.12 Data Analysis and Statistics

All results are presented as mean ± standard deviation (SD, *n* = 3). Statistical analyses were performed using one‐way ANOVA in SPSS 27, with *p* < 0.05 considered statistically significant. Graphical illustrations were prepared with GraphPad Prism 10.3 and Origin 2024.

## 3 Results

### 3.1 Screening Bioactive Components of FC Against Liver Injury

**FIGURE 2:**
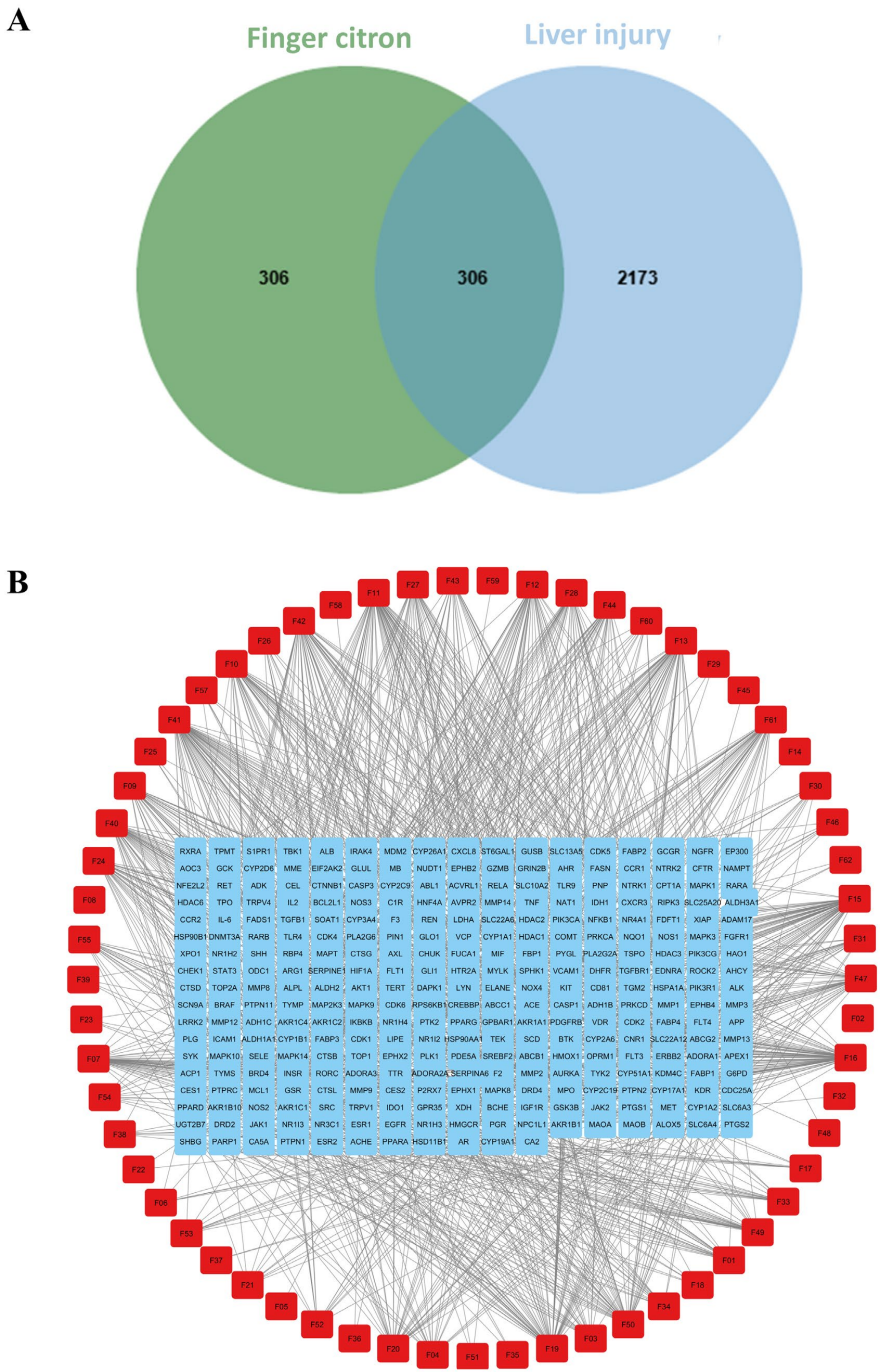
Workflow for identifying potential targets of FC against liver injury. (A) Venn diagram depicting the overlap between target genes of FC bioactive components and liver injury. (B) The interaction network of “FC bioactive components–targets–liver injury” was generated and visualized using Cytoscape 3.10.1.

We identified five potential bioactive components of FC from the TCMSP database and 139 from the HERB database. Following ADME analysis and the removal of duplicates and components lacking predicted targets, a total of 62 bioactive components were retained (Table S2). Their putative targets, predicted using the Swiss Target Prediction database, yielding 612 unique targets after removing redundancies. Additionally, genes associated with liver injury were retrieved from the OMIM, DisGeNET, and GeneCards databases. After merging and filtering these datasets, 2173 target genes were identified. The intersection of FC bioactive component targets and liver injury‐related targets was determined using Venny 2.1.0, yielding 306 overlapping genes (Figure 2A). These data were used to construct the “FC bioactive components–targets–liver injury” interaction network in Cytoscape 3.10.1 (Figure 2B). Network topology analysis based on degree values highlighted the five most connected compounds in the network were identified as 5,2′,5′‐trihydroxy‐6,7,8‐trimethoxyflavone, 5,2′,6′‐trihydroxy‐7,8‐dimethoxyflavone, diosmetin, 3,4,7‐trimethoxycoumarin, and 6,7‐dimethoxycoumarin, respectively.

### 3.2 Construction and Analysis of the PPI Network

**FIGURE 3:**
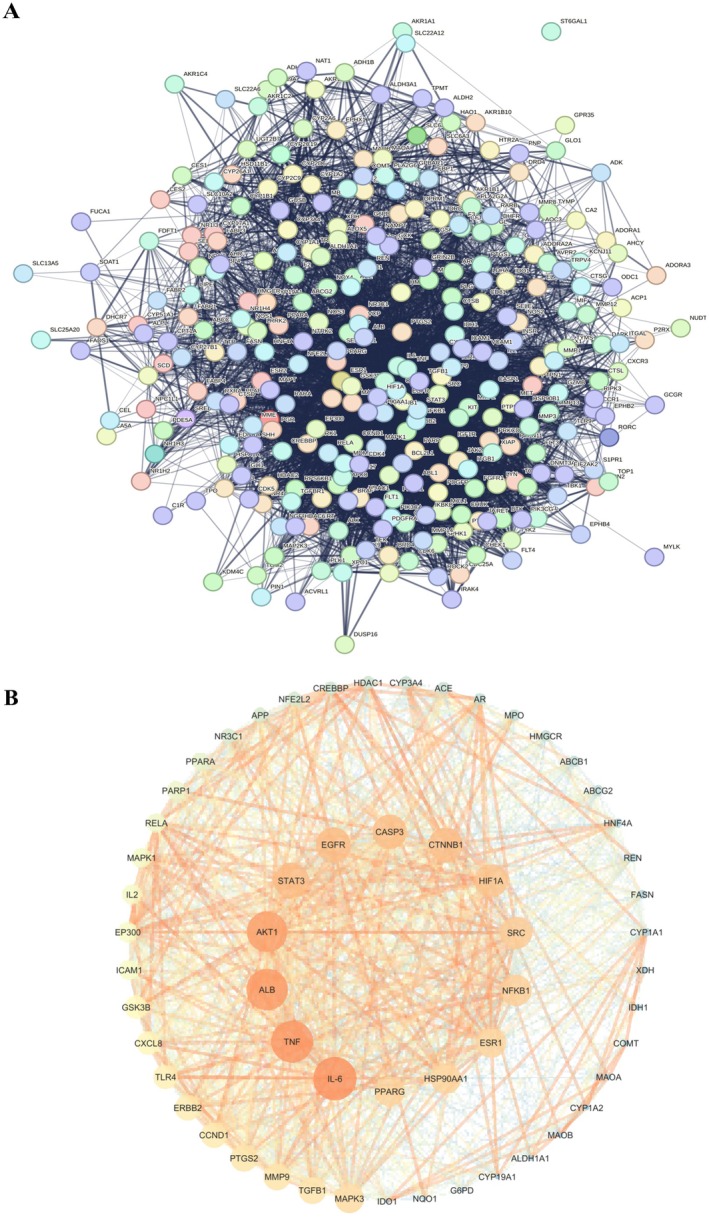
PPI network construction. (A) Interactive PPI network. (B) Network visualization in Cytoscape 3.10.1 revealed IL6, TNF‐α, ALB, AKT1, and STAT3 as the top five key targets.

The overlapping target genes of FC and liver injury were uploaded to the STRING for PPI network construction (Figure 3A). PPI data retrieved from STRING were subsequently analyzed using Cytoscape 3.10.1 (Figure 3B). In the resulting network, larger and darker nodes representing higher levels of connectivity. Based on degree value ranking, IL6, TNF‐α, ALB, AKT1, and STAT3 were identified as the top five key targets and subsequently chosen for molecular docking analysis.

### 3.3 GO and KEGG Pathway Enrichment Analysis of Intersecting Targets Between FC and Liver Injury

**FIGURE 4:**
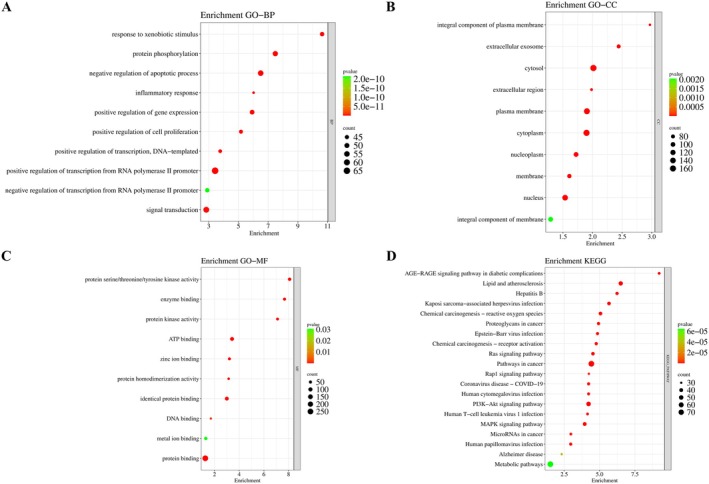
GO and KEGG pathway enrichment analysis. (A–C) GO enrichment results showing the top 10 terms for BP (A), CC (B), and MF (C) associated with the effect of FC against liver injury. (D) Sankey diagram illustrating the KEGG analysis results for the top 20 signaling pathways targeted by FC in the context of liver injury.

GO enrichment analysis identified a total of 832 biological processes (BP), 122 cellular components (CC), and 146 molecular functions (MF). The analysis focused on the top BP, CC, and MF terms (Figure 4A–C) and the top 20 KEGG pathways (Figure 4D) were selected for detailed evaluation. The findings indicate that the major BP terms primarily include protein phosphorylation, inflammatory response, apoptosis, cell proliferation, transcriptional regulation, and signal transduction. The enriched CC terms indicated that these genes are mainly localized in cellular regions such as the cytoplasm and nucleus. Moreover, MF analysis highlighted critical molecular functions including protein kinase activity, ATP binding, and protein binding. Collectively, these findings provide a deeper understanding of the molecular basis underlying the hepatoprotective effects of FC. In addition, KEGG analysis revealed that these genes were predominantly involved in the PI3K‐Akt and MAPK signaling pathways.

### 3.4 Molecular Docking Analysis

**FIGURE 5:**
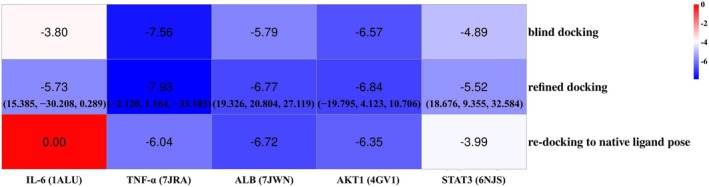
Binding energies from molecular docking and the binding coordinates from refined docking.

To elucidate the molecular interactions between TMC and the selected key targets, molecular docking analyses were conducted. Generally, a lower binding energy indicates a more stable ligand–receptor complex. As summarized in Figure 5, all five targets exhibited favorable binding affinities with TMC, suggesting their potential as molecular targets for TMC‐based NAFLD therapy. Notably, the crystal structure of IL‐6 (PDB ID: 1ALU) contained no co‐crystallized ligand.

**FIGURE 6:**
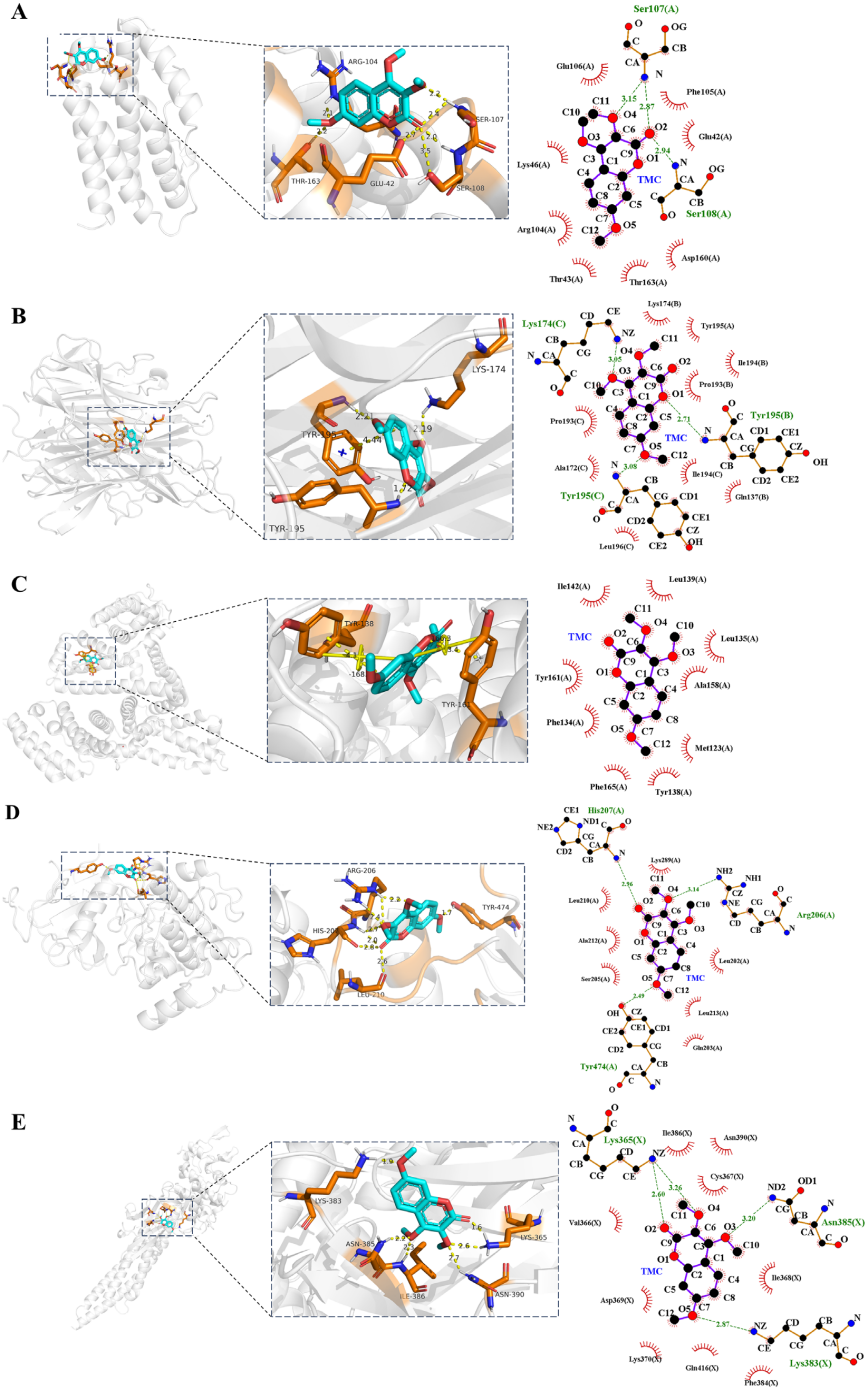
Three‐ and two‐dimensional docking models and interaction profiles between TMC and the key target proteins. (A) TMC‐IL‐6 (PDB ID: 1ALU), (B) TMC‐TNF‐α (PDB ID: 7JRA), (C) TMC‐ALB (PDB ID: 7JWN), (D) TMC‐AKT1 (PDB ID: 4GV1), (E) TMC‐STAT3 (PDB ID: 6NJS). Target interacting residues are highlighted as orange sticks.

The 3D docking models (Figure 6 and Figure S1) clearly visualize the optimal binding conformations between TMC and each target protein, revealing the formation of hydrogen bonds with several key amino acid residues. Specifically, for IL‐6, TMC formed hydrogen bonds with residues ARG104, SER107, SER108, GLU42, and THR163. In the TNF‐α complex, strong docking affinity was observed, with hydrogen bonding at TYR195 and LYS174. In ALB, hydrogen bonds were identified at TYR138 and TYR161, contributing to the stabilization of the interaction. For AKT1, key interacting residues included HIS207, ARG206, TYR474, and LEU210. In the case of STAT3, TMC formed interactions with residues LYS383, ASN385, ILE386, ASN390, and LYS365.

### 3.5 TMC Attenuates Lipid Accumulation and Oxidative Stress Stimulated by FFA

**FIGURE 7:**
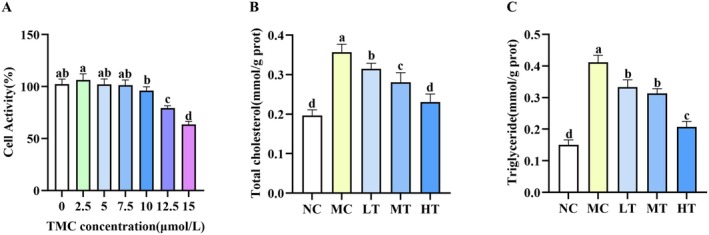
Effects of TMC on HepG2 cell viability and lipid accumulation. (A) Impact of TMC on the viability of HepG2 cells. (B, C) TMC treatment decreased the TC and TG levels in FFA‐stimulated HepG2 cells. Data are presented as mean ± SD (*n* = 3). Within each row, values labeled with identical letters (a–d) are not significantly different.

**FIGURE 8:**
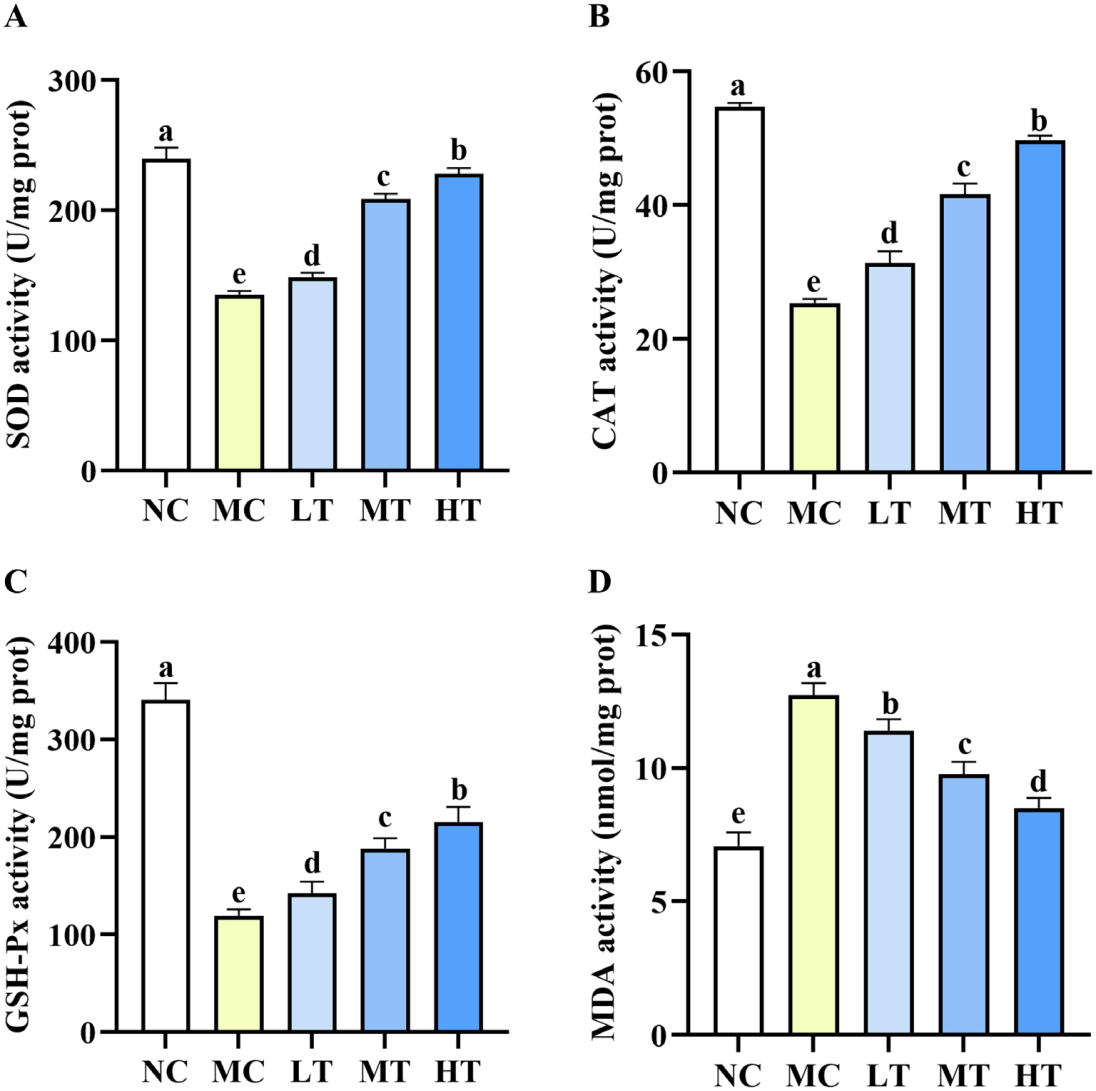
TMC alleviated oxidative stress in the FFA‐stimulated HepG2 cells. (A–C) TMC enhanced SOD, CAT, GSH‐Px activity. (D) TMC decreased MDA levels. Data are presented as mean ± SD (*n* = 3). Within each row, values labeled with identical letters (a–d) are not significantly different.

CCK‐8 assay showed that TMC concentrations up to 10 μM exhibited no significant inhibitory effect on cell growth. In contrast, concentrations exceeding 10 μM resulted in a marked reduction in cell viability (Figure 7A). Based on these observations, TMC concentrations of 5, 7.5, and 10 μM were chosen for subsequent experiments. Building on these results, we examined the ability of TMC to attenuate FFA‐stimulated lipid accumulation in HepG2 cells. As presented in Figure 7B,C, TMC treatment significantly reduced both TC and TG levels. Furthermore, the antioxidant potential of TMC was assessed. Treatment with TMC dose‐dependently restored the activities of SOD, CAT, and GSH‐Px, whereas markedly reducing MDA levels (Figure 8). These results collectively demonstrate that TMC attenuates lipid accumulation and alleviates oxidative stress.

### 3.6 The Regulation of TMC on the Relative mRNA Expression of Key Target Genes and Lipid Metabolism‐Related Genes

**FIGURE 9:**
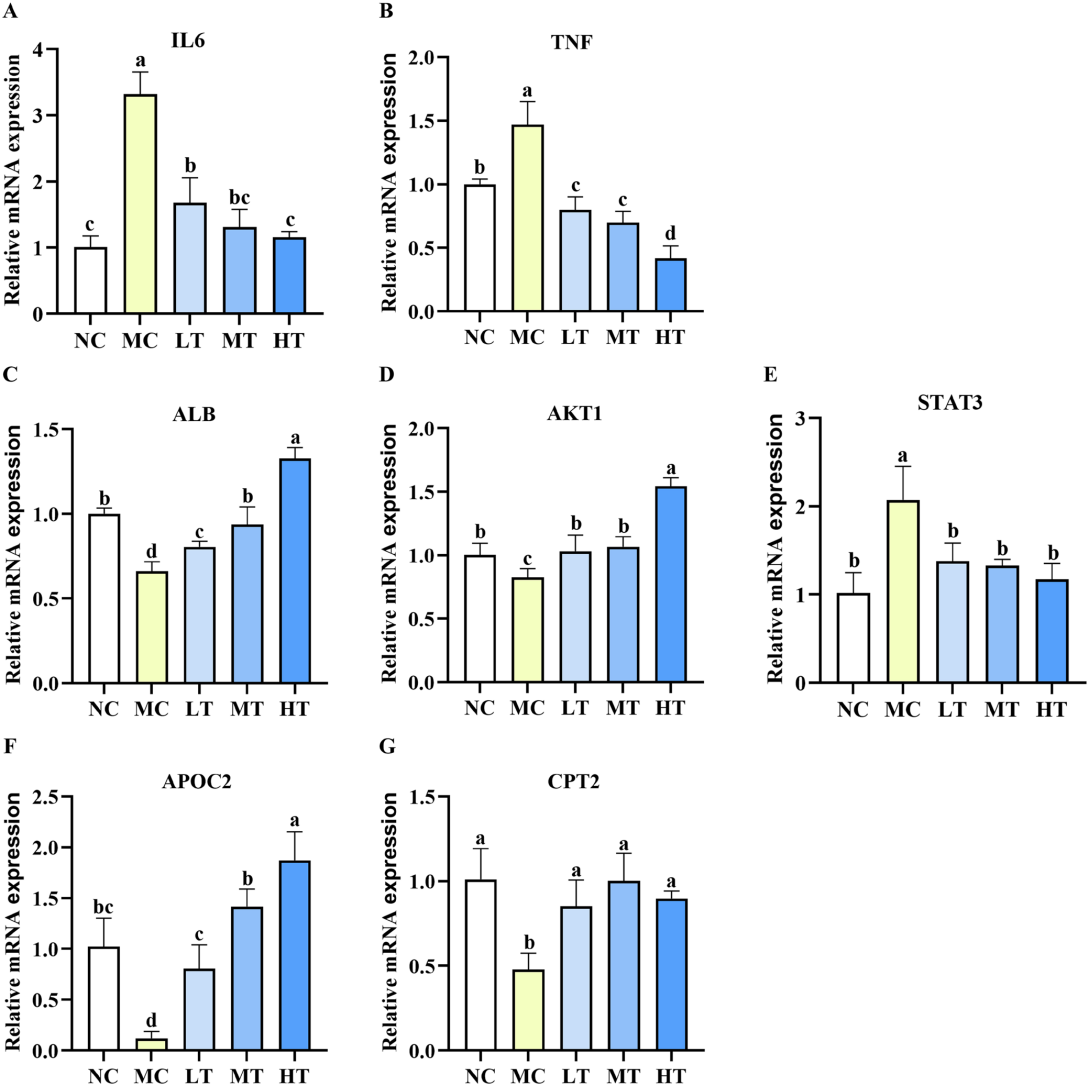
Relative mRNA expression of key target genes and lipid metabolism‐related genes in the FFA‐stimulated HepG2 cells. (A–E) TMC downregulated the expression of IL6 (A), TNF‐α (B), and STAT3 (E), whereas upregulated the expression of ALB (C) and AKT1 (D). (F, G) TMC increased the expression of lipid metabolism‐related genes APOC2 and CPT2. Data are presented as mean ± SD (*n* = 3). Within each row, values labeled with identical letters (a–d) are not significantly different.

To further substantiate the therapeutic potential of TMC against NAFLD and elucidate its association with key target genes, we quantified the relative mRNA expression of IL6, TNF‐α, ALB, AKT1, and STAT3 using RT‐qPCR. TMC treatment effectively counteracted the FFA‐stimulated upregulation of IL6、TNF‐α, and STAT3, whereas simultaneously enhancing the expression of ALB and AKT1 (Figure 9A–E).

In addition, we examined the effects of TMC on the transcriptional regulation of lipid metabolism–related genes. Notably, APOC2 was markedly reduced in the MC group following FFA exposure compared with the NC group. TMC treatment significantly upregulated the APOC2 and CPT2 (Figure 9F,G). These findings further substantiate the hypothesis that TMC attenuates lipid accumulation in FFA‐stimulated HepG2 cells.

## 4 Discussion

NAFLD stands as the predominant chronic liver condition, and represents a significant global health issue (Zhou et al. 2020). FC has been reported to modulate lipid metabolism and attenuate oxidative stress, thereby demonstrating therapeutic potential against glucolipid metabolic disorders (Yang et al. 2021; Yu et al. 2021). Nonetheless, the molecular targets and mechanisms through which FC intervenes in NAFLD have yet to be systematically elucidated. To address this question, we combined network pharmacology with molecular docking to identify the principal bioactive compounds and key targets responsible for the hepatoprotective activity of FC. These predictions were subsequently corroborated through cellular experiments, thereby creating an integrative research framework that strengthens both the reliability and robustness of our findings.

In the network pharmacology analysis, we constructed a bioactive compound–target interaction network, from which the top five candidate compounds were identified in order as follows: 5,2′,5′‐trihydroxy‐6,7,8‐trimethoxyflavone, 5,2′,6′‐trihydroxy‐7,8‐dimethoxyflavone, diosmetin, 3,4,7‐trimethoxycoumarin, and 6,7‐dimethoxycoumarin. However, due to the prohibitively high acquisition costs of the top two ranked compounds, and the extensive prior studies already conducted on the third and fifth candidates, the fourth‐ranked compound was selected for experimental validation (Luo et al. 2021; Żurawek et al. 2025; Li, Liu, et al. 2025; Zhao et al. 2024). This strategy not only ensured feasibility but also allowed exploration of a relatively understudied yet promising compound. Further PPI network analysis revealed IL6, TNF‐α, ALB, AKT1, and STAT3 as the five most prominent key targets, suggesting their critical involvement in FC‐mediated hepatoprotection. GO enrichment analysis revealed that the target genes were predominantly associated with pathways related to protein phosphorylation, inflammatory response, and protein kinase activity. Inflammatory responses primarily through the induction of oxidative stress, thereby accelerating the progression of metabolic‐associated fatty liver disease (Termite et al. 2025). Dysregulated phosphorylation of STAT3 has been linked to abnormal lipid accumulation, thereby aggravating hepatic injury (Sharma et al. 2024). In contrast, enhancing the phosphorylation of protein kinase B has been shown to improve lipid metabolism and reduce steatosis in NAFLD animal models (Pan et al. 2024). Furthermore, KEGG analysis identified the PI3K‐Akt and MAPK pathways as the key signaling cascades associated with FC's protective effects. Activation of the PI3K‐Akt pathway plays a pivotal role in modulating the expression of critical genes associated with lipid metabolism, which in turn contributes to the reduction of hepatic lipid deposition in vivo (Chu et al. 2022). In contrast, overactivation of the MAPK pathway has been linked to increased reactive oxygen species generation, amplifying oxidative stress and cellular damage in hepatocytes (Li, Kuang, et al. 2025).

Our molecular docking study confirms the hepatoprotective potential of TMC, the key bioactive compound of FC. Refined docking simulations revealed that the binding free energies between TMC and the five key target proteins ranged from −5.52 to −7.93 kcal/mol, indicating favorable binding affinities. Among these, TMC exhibited the strongest interaction with TNF‐α, showing a binding energy of −7.93 kcal/mol, suggesting the formation of a particularly stable ligand–receptor complex. The interactions between TMC and its targets were primarily mediated by hydrogen bonding, π–π stacking, and other essential non‐covalent interactions including electrostatic and hydrophobic forces. These results offer mechanistic insights into the hepatoprotective effects of FC, revealed through an integrative, data mining–driven approach.

To our current understanding, this study offers the first direct in vitro evidence that TMC can reduce lipid accumulation and mitigate oxidative stress in HepG2 cells. Apolipoprotein C‐II, encoded by the APOC2 gene, facilitates the hydrolysis and clearance of TG (Gu et al. 2022; Wu et al. 2024). Excessive lipid deposition is shown to impair mitochondrial function, thereby disrupting fatty acid β‐oxidation and cellular energy metabolism, which in turn exacerbates the intracellular fatty acid accumulation (Carotti et al. 2020). Meanwhile, CPT2 is essential for mitochondrial fatty acid β‐oxidation, a critical pathway in lipid catabolism (Xu et al. 2024). In this study, TMC treatment significantly upregulated APOC2 and CPT2 expression, whereas markedly decreased intracellular TC and TG levels. These findings suggest that TMC exerts a lipid‐lowering effect by modulating key genes involved in lipid metabolism. Oxidative stress is widely recognized as a central contributor to NAFLD pathogenesis, and its suppression is regarded as a fundamental therapeutic strategy for both prevention and treatment (Arroyave‐Ospina et al. 2021). SOD represents the primary defense against oxidative damage by catalyzing the dismutation of superoxide anion radicals into hydrogen peroxide (H_2_O_2_) and molecular oxygen (Li et al. 2021). Subsequently, H_2_O_2_ is detoxified predominantly through the enzymatic actions of CAT and GSH‐Px, the latter utilizing reduced glutathione as a substrate (Zhao et al. 2019; Aleksunes and Manautou 2007). In this study, TMC treatment significantly elevated the activities of these key antioxidant enzymes, whereas simultaneously reducing MDA levels, a biomarker of lipid peroxidation. These results suggest that TMC effectively attenuates FFA‐stimulated oxidative stress in HepG2 cells, thereby contributing to its therapeutic potential role against NAFLD.

We further validated the predictions derived from network pharmacology. The results demonstrated that TMC ameliorates NAFLD by modulating the mRNA expression of key target genes. In NAFLD, inflammatory responses are marked by elevated levels of pro‐inflammatory cytokines such as IL‐6 and TNF‐α (Giuffrè et al. 2024). IL‐6 has been shown to induce aberrant phosphorylation of STAT3, which perturbs lipid metabolism, thereby accelerating disease progression (Shao et al. 2020). TNF‐α activates the MAPK signaling cascade, promoting excessive production of inflammatory mediators and increasing oxidative stress in hepatocytes (Li, Liang, et al. 2025). AKT1 is the central kinase in the downstream segment of the PI3K‐Akt pathway, plays a crucial role in promoting fatty acid oxidation, reducing lipid accumulation, and ameliorate NAFLD in murine models (Sun et al. 2023). Additionally, ALB, a marker of hepatic function, is regulated by PI3K‐Akt signaling; decreased ALB expression is indicative of impaired hepatocellular function (Sun et al. 2019). Collectively, these findings suggest that TMC downregulates the upstream regulators of PI3K‐Akt and MAPK pathways, such as IL6 and TNF‐α, along with the key downstream kinase AKT1, thereby attenuating lipid accumulation and oxidative stress. The observed increase in ALB expression further reflects the restoration of HepG2 cell function and supports the therapeutic potential of TMC in NAFLD.

Therefore, our study not only identified the bioactive compounds and key targets underlying the hepatoprotective effects of FC through network pharmacology, but also further validated these targets via bioinformatics‐based molecular docking. Ultimately, we confirmed the therapeutic effect of TMC, a representative active constituent of FC, in ameliorating NAFLD through in vitro cellular experiments. Although cell‐based assays cannot fully recapitulate the pathological complexity of NAFLD in vivo, our results provide meaningful mechanistic insights and robust preliminary evidence supporting the therapeutic potential of TMC in NAFLD prevention and treatment. Nevertheless, further investigations—particularly in vivo studies and clinical validations—are warranted to substantiate these findings.

## 5 Conclusions

In conclusion, building upon predictions derived from network pharmacology, we applied molecular docking together with in vitro experiments to systematically validate the interactions between TMC—the principal bioactive constituent of FC—and its key targets, whereas also confirming its ameliorative effects and underlying mechanisms against NAFLD. The results indicate that TMC markedly reduces lipid accumulation and oxidative stress in hepatocytes, thereby exerting anti‐NAFLD effects. Our findings lay a scientific foundation for the targeted development and application of FC in functional foods.

## Author Contributions

**Qingpeng Li:** conceptualization, methodology, data curation, investigation, validation, formal analysis, visualization, writing – original draft. **Huilong Sun:** conceptualization, methodology, software, data curation, investigation, formal analysis, writing – original draft. **Xiaoling Chen:** conceptualization, methodology, software, validation, formal analysis, visualization, writing – original draft. **Chunyang Chen:** methodology, data curation, investigation, validation, resources, writing – original draft. **Yongsheng Chen:** software, investigation, supervision, visualization, resources, writing – original draft. **Hui He:** methodology, software, investigation, validation, visualization, writing – original draft. **Fan Yang:** investigation, formal analysis, supervision, visualization, project administration, writing – review and editing. **Dan Wang:** methodology, software, validation, supervision, visualization, resources, writing – review and editing. **Lin Zhou:** conceptualization, methodology, formal analysis, supervision, funding acquisition, project administration, resources, writing – review and editing.

## Funding

This research was funded by grants from National Key Research and Development Program of China (No. 2023YFC3606200), National Natural Science Foundation of China (No. 82274357).

## Conflicts of Interest

The authors declare no conflicts of interest.

## Supporting information


**Figure S1:** Electrostatic surface maps of the binding pockets between TMC and the key target proteins.
**Table S1:** The sequence of primers.
**Table S2:** Bioactive components of FC.

## Data Availability

The data that support the findings of this study are available from the corresponding author upon reasonable request.
