# Supplementary material for: Integrative Network Pharmacology, Molecular Docking, and In Vitro Insights Into the Mechanism of Finger Citron for Non‐Alcoholic Fatty Liver Disease
Source: Food Sci Nutr. 2026 Apr 3;14(4):e71695. doi: 10.1002/fsn3.71695 (PMC13052329; doi:10.1002/fsn3.71695)
Supplement: Supplementary file 1 — Figure S1: Electrostatic surface maps of the binding pockets between TMC and the key target proteins. Table S1: The sequence of primers. Table S2: Bioactive components of FC. [file FSN3-14-e71695-s001.zip › Supplementary Material Presentation/Supplementary Material.docx]

Supplementary Material

# Supplementary Figures and Tables

# Supplementary Figures


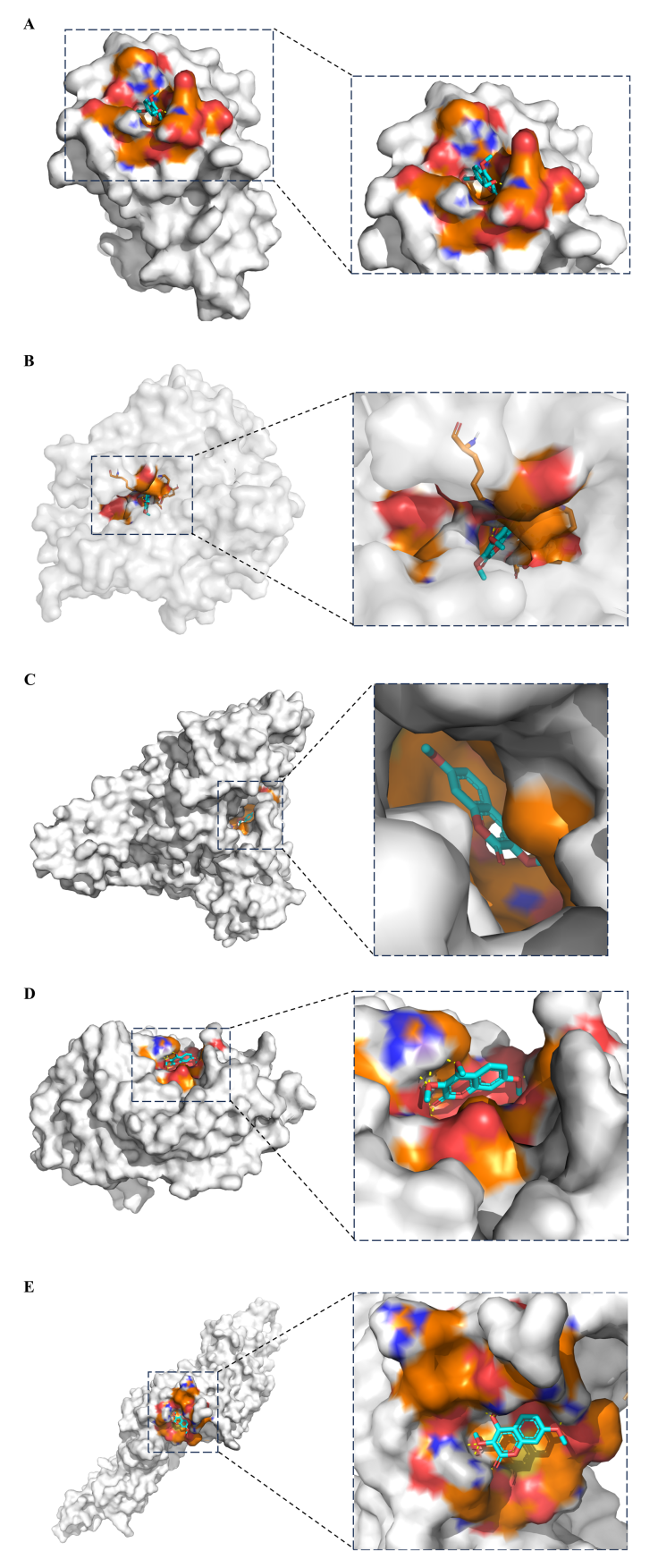


**Figure S1.** Electrostatic surface maps of the binding pockets between TMC and the key target proteins. (A) TMC–IL-6, (B) TMC–TNF-α, (C) TMC–ALB, (D) TMC–AKT1, (E) TMC–STAT3.

## Supplementary Tables

**Table S1.** The sequence of primers.

| **Gene** | **Primer Sequences（5'-3'）** |
| --- | --- |
| IL6 forward | ACTCACCTCTTCAGAACGAATTG |
| IL6 reverse | CCATCTTTGGAAGGTTCAGGTTG |
| TNF forward | CCTCTCTCTAATCAGCCCTCTG |
| TNF reverse | GAGGACCTGGGAGTAGATGAG |
| ALB forward | GAGACCAGAGGTTGATGTGATG |
| ALB reverse | AGTTCCGGGGCATAAAAGTAAG |
| AKT1 forward | TCCTCCTCAAGAATGATGGCA |
| AKT1 reverse | GTGCGTTCGATGACAGTGGT |
| STAT3 forward | ATCACGCCTTCTACAGACTGC |
| STAT3 reverse | CATCCTGGAGATTCTCTACCACT |
| CPT2 forward | CATACAAGCTACATTTCGGGACC |
| CPT2 reverse | AGCCCGGAGTGTCTTCAGAA |
| APOC2 forward | TGTCCTCCTGGTATTGGGATTT |
| APOC2 reverse | TGTCTTCTCGTACAGGTTCTGG |
| GAPDH forward | GAGTCAACGGATTTGGTCGT |
| GAPDH reverse | GACAAGCTTCCCGTTCTCAG |

**Table S2.** Bioactive components of FC.

| **NO.** | **Ingredient ID** | **Component name** | **Formula** | **Inchikey** |
| --- | --- | --- | --- | --- |
| F01 | [HBIN000772](http://herb.ac.cn/Detail/?v=HBIN000772&label=Ingredient) | 1-methyl-4-(1-methylethyl)-1,2-cyclohexanediol | [C10H20O2](https://pubchem.ncbi.nlm.nih.gov/#query=C10H20O2) | RYLFWQILVNWPEL-UHFFFAOYSA-N |
| F02 | [HBIN001519](http://herb.ac.cn/Detail/?v=HBIN001519&label=Ingredient) | 1-(4-methoxyphenyl)-2-phenyle thane | [C15H16O](https://pubchem.ncbi.nlm.nih.gov/#query=C15H16O) | AFPZIAQJQAQDAQ-UHFFFAOYSA-N |
| F03 | [HBIN002064](http://herb.ac.cn/Detail/?v=HBIN002064&label=Ingredient) | 3,7-dimethyl-1,7-octadien-3-ol | [C10H18O](https://pubchem.ncbi.nlm.nih.gov/#query=C10H18O) | GYJHTGZQPKPEOT-UHFFFAOYSA-N |
| F04 | HBIN003139 | (1R,4S,5S)-1-isopropyl-4-methylbicyclo[3.1.0]hexan-2-ol | [C10H18O](https://pubchem.ncbi.nlm.nih.gov/#query=C10H18O) | KXSDPILWMGFJMM-KXUCPTDWSA-N |
| F05 | [HBIN003949](http://herb.ac.cn/Detail/?v=HBIN003949&label=Ingredient) | meso-2,3-butanediol | [C4H10O2](https://pubchem.ncbi.nlm.nih.gov/#query=C4H10O2) | OWBTYPJTUOEWEK-UHFFFAOYSA-N |
| F06 | [HBIN003956](http://herb.ac.cn/Detail/?v=HBIN003956&label=Ingredient) | 2,3-didehydro-1,3,3-trimethyl-2-oxabicyclo[2.2.2]octa-ne | [C10H16O](https://pubchem.ncbi.nlm.nih.gov/#query=C10H16O) | LOOYOTLEOHYYOV-UHFFFAOYSA-N |
| F07 | [HBIN004217](http://herb.ac.cn/Detail/?v=HBIN004217&label=Ingredient) | 2,4,4-trimethyl-3-(3-oxobutyl)cyclohex-2-en-1-one | [C13H20O2](https://pubchem.ncbi.nlm.nih.gov/#query=C13H20O2) | QUHPCHDTFDSTTL-UHFFFAOYSA-N |
| F08 | HBIN004827 | 2,6,10-trimethyldodecane | [C6H8O4](https://pubchem.ncbi.nlm.nih.gov/#query=C6H8O4) | VOLMSPGWNYJHQQ-UHFFFAOYSA-N |
| F09 | [HBIN006274](http://herb.ac.cn/Detail/?v=HBIN006274&label=Ingredient) | 7-butyl-2-oxepanone | [C10H18O2](https://pubchem.ncbi.nlm.nih.gov/#query=C10H18O2) | YKVIWISPFDZYOW-UHFFFAOYSA-N |
| F10 | HBIN006705 | [(2S,3S)-3-methyl-3-(4-methylpent-3-enyl)oxiran-2-yl]methyl acetate | [C12H20O3](https://pubchem.ncbi.nlm.nih.gov/#query=C12H20O3) | HUUKHKAFVDAFIX-RYUDHWBXSA-N |
| F11 | [HBIN007321](http://herb.ac.cn/Detail/?v=HBIN007321&label=Ingredient) | 3,4,7-trimethoxycoumarin | [C12H12O5](https://pubchem.ncbi.nlm.nih.gov/#query=C12H12O5) | AYMSWLVOSROJFX-UHFFFAOYSA-N |
| F12 | [HBIN009499](http://herb.ac.cn/Detail/?v=HBIN009499&label=Ingredient) | (3R)-3,7-dimethyloct-6-en-1-yl acetate | [C12H22O2](https://pubchem.ncbi.nlm.nih.gov/#query=C12H22O2) | JOZKFWLRHCDGJA-LLVKDONJSA-N |
| F13 | [HBIN009844](http://herb.ac.cn/Detail/?v=HBIN009844&label=Ingredient) | (1R)-4-[2,6,6-trimethylcyclohex-2-en-1-yl]butan-2-one | [C13H22O](https://pubchem.ncbi.nlm.nih.gov/#query=C13H22O) | JHJCHCSUEGPIGE-UHFFFAOYSA-N |
| F14 | [HBIN010390](http://herb.ac.cn/Detail/?v=HBIN010390&label=Ingredient) | 4-ethyl-1,2-dimethylbenzene | [C10H14](https://pubchem.ncbi.nlm.nih.gov/#query=C10H14) | SBUYFICWQNHBCM-UHFFFAOYSA-N |
| F15 | MOL013253 | 2',5,5'-trihydroxy-6,7,8-trimethoxyflavone | [C18H16O8](https://pubchem.ncbi.nlm.nih.gov/#query=C18H16O8) | PVSHCMCDBFRZFY-UHFFFAOYSA-N |
| F16 | MOL002917 | 2',5,6'-trihydroxy-7,8-dimethoxyflavone | [C23H22O13](https://pubchem.ncbi.nlm.nih.gov/#query=C23H22O13) | GWRFNPJGKCUUSJ-UHFFFAOYSA-N |
| F17 | [HBIN011726](http://herb.ac.cn/Detail/?v=HBIN011726&label=Ingredient) | 5-isopropyl-2-methylbicyclo[3.1.0]hex-2-ene | [C10H16](https://pubchem.ncbi.nlm.nih.gov/#query=C10H16) | KQAZVFVOEIRWHN-UHFFFAOYSA-N |
| F18 | [HBIN012057](http://herb.ac.cn/Detail/?v=HBIN012057&label=Ingredient) | 6,7-dihydrogeraniol | [C10H20O](https://pubchem.ncbi.nlm.nih.gov/#query=C10H20O) | JNAWJUOCXKIHHG-JXMROGBWSA-N |
| F19 | [HBIN012080](http://herb.ac.cn/Detail/?v=HBIN012080&label=Ingredient) | 6,7-dimethoxycoumarin | [C11H10O4](https://pubchem.ncbi.nlm.nih.gov/#query=C11H10O4) | GUAFOGOEJLSQBT-UHFFFAOYSA-N |
| F20 | [HBIN013253](http://herb.ac.cn/Detail/?v=HBIN013253&label=Ingredient) | 7-hydroxy-6-methoxy-coumarin | [C10H8O4](https://pubchem.ncbi.nlm.nih.gov/#query=C10H8O4) | RODXRVNMMDRFIK-UHFFFAOYSA-N |
| F21 | [HBIN014406](http://herb.ac.cn/Detail/?v=HBIN014406&label=Ingredient) | Acetic acid, bornyl ester | [C12H20O2](https://pubchem.ncbi.nlm.nih.gov/#query=C12H20O2) | KGEKLUUHTZCSIP-UHFFFAOYSA-N |
| F22 | [HBIN014419](http://herb.ac.cn/Detail/?v=HBIN014419&label=Ingredient) | Acetol | [C3H6O2](https://pubchem.ncbi.nlm.nih.gov/#query=C3H6O2) | XLSMFKSTNGKWQX-UHFFFAOYSA-N |
| F23 | [HBIN015441](http://herb.ac.cn/Detail/?v=HBIN015441&label=Ingredient) | α-caryophyllene | [C15H24](https://pubchem.ncbi.nlm.nih.gov/#query=C15H24) | FAMPSKZZVDUYOS-HRGUGZIWSA-N |
| F24 | [HBIN015457](http://herb.ac.cn/Detail/?v=HBIN015457&label=Ingredient) | α-citronellal | [C10H18O](https://pubchem.ncbi.nlm.nih.gov/#query=C10H18O) | KQZAHMIZMBERJX-UHFFFAOYSA-N |
| F25 | [HBIN015645](http://herb.ac.cn/Detail/?v=HBIN015645&label=Ingredient) | α-phellandrene | [C10H16](https://pubchem.ncbi.nlm.nih.gov/#query=C10H16) | OGLDWXZKYODSOB-UHFFFAOYSA-N |
| F26 | [HBIN015652](http://herb.ac.cn/Detail/?v=HBIN015652&label=Ingredient) | α-pinene | [C10H16](https://pubchem.ncbi.nlm.nih.gov/#query=C10H16) | GRWFGVWFFZKLTI-RKDXNWHRSA-N |
| F27 | [HBIN015704](http://herb.ac.cn/Detail/?v=HBIN015704&label=Ingredient) | α-terpineol | [C10H18O](https://pubchem.ncbi.nlm.nih.gov/#query=C10H18O) | WUOACPNHFRMFPN-VIFPVBQESA-N |
| F28 | [HBIN017418](http://herb.ac.cn/Detail/?v=HBIN017418&label=Ingredient) | Ayapanin | [C10H8O3](https://pubchem.ncbi.nlm.nih.gov/#query=C10H8O3) | LIIALPBMIOVAHH-UHFFFAOYSA-N |
| F29 | [HBIN017658](http://herb.ac.cn/Detail/?v=HBIN017658&label=Ingredient) | β-cubebene | [C15H24](https://pubchem.ncbi.nlm.nih.gov/#query=C15H24) | FSRZGYRCMPZNJF-KHMAMNHCSA-N |
| F30 | [HBIN018023](http://herb.ac.cn/Detail/?v=HBIN018023&label=Ingredient) | β-citral | [C10H16O](https://pubchem.ncbi.nlm.nih.gov/#query=C10H16O) | WTEVQBCEXWBHNA-YFHOEESVSA-N |
| F31 | [HBIN018178](http://herb.ac.cn/Detail/?v=HBIN018178&label=Ingredient) | β-linalool | C10H18O | DOSHBSSFJOMGT-UHFFFAOYSA-N |
| F32 | [HBIN018346](http://herb.ac.cn/Detail/?v=HBIN018346&label=Ingredient) | β-trans-ocimene | [C10H16](https://pubchem.ncbi.nlm.nih.gov/#query=C10H16) | IHPKGUQCSIINRJ-CSKARUKUSA-N |
| F33 | HBIN018427 | 2,7,7-trimethylbicyclo[3.1.1]hept-2-en-6-yl acetate | [C12H18O2](https://pubchem.ncbi.nlm.nih.gov/#query=C12H18O2) | UASZOTVHPVEMQR-AXFHLTTASA-N |
| F34 | [HBIN018462](http://herb.ac.cn/Detail/?v=HBIN018462&label=Ingredient) | 5,5-dimethylbicyclo[6.3.0]undeca-1,7-dien-3-one | [C13H18O](https://pubchem.ncbi.nlm.nih.gov/#query=C13H18O) | NNVYWFNVUGKOGN-XNFNPUBGSA-N |
| F35 | [HBIN018464](http://herb.ac.cn/Detail/?v=HBIN018464&label=Ingredient) | 4,8,11,11-tetramethylbicyclo[7.2.0]undec-4-ene |  |  |
| F36 | HBIN019817 | Caryophyllene | [C15H24](https://pubchem.ncbi.nlm.nih.gov/#query=C15H24) | FAMPSKZZVDUYOS-HRGUGZIWSA-N |
| F37 | [HBIN019825](http://herb.ac.cn/Detail/?v=HBIN019825&label=Ingredient) | Caryophyllene oxide | [C15H24O](https://pubchem.ncbi.nlm.nih.gov/#query=C15H24O) | NVEQFIOZRFFVFW-RGCMKSIDSA-N |
| F38 | [HBIN020749](http://herb.ac.cn/Detail/?v=HBIN020749&label=Ingredient) | Cis-2,8-menthadien-1-ol | [C10H16O](https://pubchem.ncbi.nlm.nih.gov/#query=C10H16O) | MKPMHJQMNACGDI-UWVGGRQHSA-N |
| F39 | [HBIN020877](http://herb.ac.cn/Detail/?v=HBIN020877&label=Ingredient) | Cis-limonene oxide | [C10H16O](https://pubchem.ncbi.nlm.nih.gov/#query=C10H16O) | CCEFMUBVSUDRLG-KXUCPTDWSA-N |
| F40 | [HBIN021002](http://herb.ac.cn/Detail/?v=HBIN021002&label=Ingredient) | Citropten | [C11H10O4](https://pubchem.ncbi.nlm.nih.gov/#query=C11H10O4) | NXJCRELRQHZBQA-UHFFFAOYSA-N |
| F41 | MOL002881 | [Diosmetin](https://www.tcmsp-e.com/molecule.php?qn=2881) | [C16H12O6](https://pubchem.ncbi.nlm.nih.gov/#query=C16H12O6) | MBNGWHIJMBWFHU-UHFFFAOYSA-N |
| F42 | [HBIN024194](http://herb.ac.cn/Detail/?v=HBIN024194&label=Ingredient) | Diosphenol | [C10H16O2](https://pubchem.ncbi.nlm.nih.gov/#query=C10H16O2) | QSIMLPCPCXVYDD-UHFFFAOYSA-N |
| F43 | [HBIN027529](http://herb.ac.cn/Detail/?v=HBIN027529&label=Ingredient) | Geraniol acetate | [C11H18O2](https://pubchem.ncbi.nlm.nih.gov/#query=C11H18O2) | FMFCRRDJQXJLBD-CSKARUKUSA-N |
| F44 | [HBIN029328](http://herb.ac.cn/Detail/?v=HBIN029328&label=Ingredient) | Hexanoic acid | [C6H12O2](https://pubchem.ncbi.nlm.nih.gov/#query=C6H12O2) | FUZZWVXGSFPDMH-UHFFFAOYSA-N |
| F45 | [HBIN029706](http://herb.ac.cn/Detail/?v=HBIN029706&label=Ingredient) | Hydroxybutyric acid- | [C4H8O3](https://pubchem.ncbi.nlm.nih.gov/#query=C4H8O3) | SJZRECIVHVDYJC-UHFFFAOYSA-N |
| F46 | [HBIN030615](http://herb.ac.cn/Detail/?v=HBIN030615&label=Ingredient) | Isocoumarin | [C9H6O2](https://pubchem.ncbi.nlm.nih.gov/#query=C9H6O2) | IQZZFVDIZRWADY-UHFFFAOYSA-N |
| F47 | [HBIN030735](http://herb.ac.cn/Detail/?v=HBIN030735&label=Ingredient) | Isoferulic acid | [C10H10O4](https://pubchem.ncbi.nlm.nih.gov/#query=C10H10O4) | QURCVMIEKCOAJU-HWKANZROSA-N |
| F48 | [HBIN032234](http://herb.ac.cn/Detail/?v=HBIN032234&label=Ingredient) | Kojic acid | [C6H6O4](https://pubchem.ncbi.nlm.nih.gov/#query=C6H6O4) | BEJNERDRQOWKJM-UHFFFAOYSA-N |
| F49 | [HBIN032467](http://herb.ac.cn/Detail/?v=HBIN032467&label=Ingredient) | L-4-terpineol | [C10H18O](https://pubchem.ncbi.nlm.nih.gov/#query=C10H18O) | WRYLYDPHFGVWKC-JTQLQIEISA-N |
| F50 | [HBIN032754](http://herb.ac.cn/Detail/?v=HBIN032754&label=Ingredient) | Lauric acid | [C12H24O2](https://pubchem.ncbi.nlm.nih.gov/#query=C12H24O2) | POULHZVOKOAJMA-UHFFFAOYSA-N |
| F51 | [HBIN033428](http://herb.ac.cn/Detail/?v=HBIN033428&label=Ingredient) | L-limonen | [C10H16](https://pubchem.ncbi.nlm.nih.gov/#query=C10H16) | XMGQYMWWDOXHJM-SNVBAGLBSA-N |
| F52 | [HBIN035690](http://herb.ac.cn/Detail/?v=HBIN035690&label=Ingredient) | Monopalmitin | [C19H38O4](https://pubchem.ncbi.nlm.nih.gov/#query=C19H38O4) | QHZLMUACJMDIAE-UHFFFAOYSA-N |
| F53 | HBIN036795 | 3,7-dimethyl-2,6-octadien-1-ol | [C10H18O](https://pubchem.ncbi.nlm.nih.gov/#query=C10H18O) | GLZPCOQZEFWAFX-YFHOEESVSA-N |
| F54 | [HBIN040908](http://herb.ac.cn/Detail/?v=HBIN040908&label=Ingredient) | Protocatechuic acid | [C7H5O4](https://pubchem.ncbi.nlm.nih.gov/#query=C7H5O4-) | YQUVCSBJEUQKSH-UHFFFAOYSA-M |
| F55 | [HBIN043423](http://herb.ac.cn/Detail/?v=HBIN043423&label=Ingredient) | (S)-cis-verbenol | [C10H16O](https://pubchem.ncbi.nlm.nih.gov/#query=C10H16O) | WONIGEXYPVIKFS-YIZRAAEISA-N |
| F56 | [HBIN043437](http://herb.ac.cn/Detail/?v=HBIN043437&label=Ingredient) | Scoparone | [C11H10O4](https://pubchem.ncbi.nlm.nih.gov/#query=C11H10O4) | GUAFOGOEJLSQBT-UHFFFAOYSA-N |
| F57 | HBIN043798 | Sesamin | [C20H18O6](https://pubchem.ncbi.nlm.nih.gov/#query=C20H18O6) | PEYUIKBAABKQKQ-AFHBHXEDSA-N |
| F58 | MOL001506 | [Supraene](https://www.tcmsp-e.com/molecule.php?qn=1506) | [C30H50](https://pubchem.ncbi.nlm.nih.gov/#query=C30H50) | YYGNTYWPHWGJRM-AAJYLUCBSA-N |
| F59 | [HBIN045937](http://herb.ac.cn/Detail/?v=HBIN045937&label=Ingredient) | Tereben | [C10H16](https://pubchem.ncbi.nlm.nih.gov/#query=C10H16) | MOYAFQVGZZPNRA-UHFFFAOYSA-N |
| F60 | HBIN047738 | Vanillic acid | [C8H8O4](https://pubchem.ncbi.nlm.nih.gov/#query=C8H8O4) | WKOLLVMJNQIZCI-UHFFFAOYSA-N |
| F61 | [HBIN048942](http://herb.ac.cn/Detail/?v=HBIN048942&label=Ingredient) | (-)-epi-α-bisabolol | C15H26O | GZSQWQPBWRIAQ-PVRQQBJHNA-N |
| F62 | [HBIN049168](http://herb.ac.cn/Detail/?v=HBIN049168&label=Ingredient) | γ-terpinene | [C10H16](https://pubchem.ncbi.nlm.nih.gov/#query=C10H16) | YKFLAYDHMOASIY-UHFFFAOYSA-N |
